# Adsorption of iron tetraphenylporphyrin on (111) surfaces of coinage metals: a density functional theory study

**DOI:** 10.3762/bjnano.8.248

**Published:** 2017-11-23

**Authors:** Hao Tang, Nathalie Tarrat, Véronique Langlais, Yongfeng Wang

**Affiliations:** 1 CEMES/CNRS, 29 rue Jeanne Marvig, P.O. Box 94347, 31055 Toulouse CEDEX 4, France; 2 Key Laboratory for the Physics and Chemistry of Nanodevices, Department of Electronics, Peking University, Beijing 100871, China

**Keywords:** activation barrier, density functional theory, iron tetraphenylporphyrin, spin switch, spin states

## Abstract

The adsorption of the iron tetraphenylporphyrin (FeTPP) molecule in its deckchair conformation was investigated on Au(111), Ag(111) and Cu(111) surfaces by performing spin-polarized density functional theory (DFT) calculations taking into account both van der Waals (vdW) interaction and on-site Coulomb repulsion. The deckchair conformation of the molecule favours intermolecular π–π-type interactions in a less densely packed monolayer than the saddle conformation. The activation barrier between the two stable magnetic states (high spin, *S* = 2 and intermediate spin, *S* = 1) of the molecule in vacuum disappears upon adsorption on the metal surfaces. The high-spin state of physisorbed FeTPP is stable on all adsorption sites. This result reveals that an external permanent element such as a STM tip or an additional molecule is needed to use FeTPP or similar molecules as model system for molecular spin switches.

## Introduction

Porphyrins, phthalocyanines and their transition-metal (TM) complexes are largely investigated in surface science as reported in detail by Gottfried [1]. The nature of the central metal atom greatly determines the electronic, magnetic, catalytic properties of these molecules. Once adsorbed on metallic surfaces, these properties could be significantly modified due to the interaction between the central macrocycle of these molecules and the substrate. Among these complexes, iron tetraphenylporphyrin (FeTPP) is particularly attractive for molecular spintronics due to its magnetic bistability. Indeed, the Fe^2+^ centre (4s^0^3d^6^) can have three magnetic states, i.e., low-spin state (LS, *S* = 0), intermediate state (IS, *S* = 1) and high-spin state (HS, *S* = 2). While the LS ground state is mostly observed in sixfold-coordinated molecular complexes, the ground state of square planar fourfold-coordinated Fe porphyrin can be either IS or HS depending on the functional groups, characterization method or approximation used [2–7]. The main difference between these two stable magnetic states is essentially associated to the strength of the ligand field. The modification of the coordination sphere of the metallic centre is thus necessary to manipulate its spin state. Most of such manipulations have been achieved by coordinating an additional small molecule (e.g., NO, CO) or atom (Cl) in order to modify the coordination number [8–11]. Only few reversible manipulations of spin were achieved without additional ligand, as the one shown by N. Lin et al. on a single FeTPP molecule junction in a scanning tunnelling microscope (STM) [12]. In this junction, the line shape of zero-bias resonance of the adsorbed FeTPP molecule reversibly varies by adjusting the tip to surface distance, i.e., by mechanically squeezing the molecule. Density functional theory (DFT) calculations reveal that the spin state of the Fe centre undergoes a switch from *S* = 2 to *S* = 1 associated with a conformational change by passing from a saddle shape to a planar shape in the presence of the STM tip.

To the best of our knowledge, the stability as well as the activation barrier between HS and IS FeTPP have not yet been investigated. In this paper, a brief analysis of the free FeTPP molecule conformations is presented at first, together with an evaluation of the activation barrier between these conformations. The magnetic switch barrier is then evaluated for the *C*_2_*_h_* conformation. Second, the monolayer of this molecule on (111) surfaces of Au, Ag and Cu is investigated. At the end, the relationship between the substrate and the coordination sphere of Fe is discussed in terms of molecule–surface interaction, charge transfer and work function modification.

## Results and Discussion

### Free molecule and conformations

**Figure 1:**
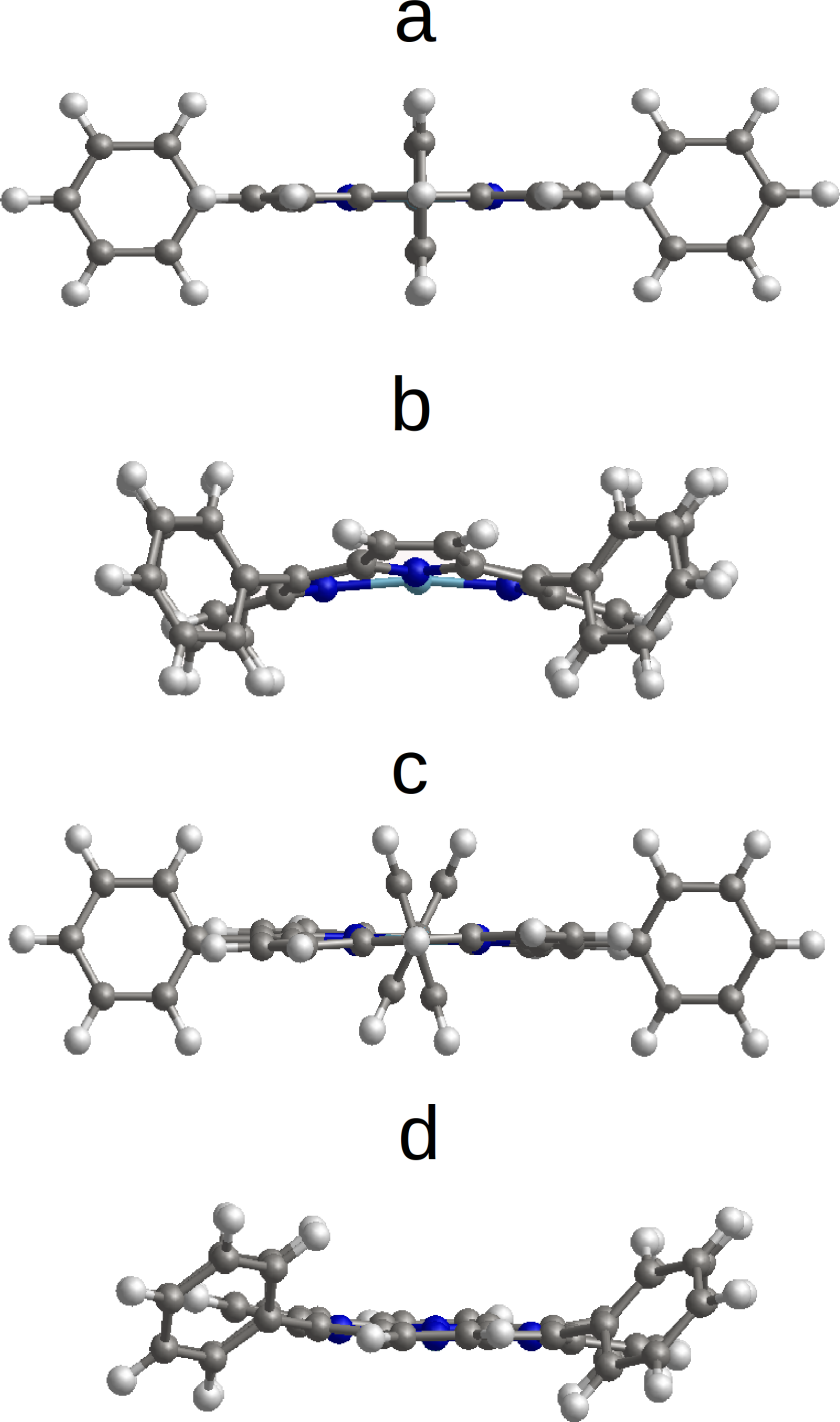
Low-symmetry FeTPP conformations: a) ideal (*D*_4_*_h_*); b) saddle (*D*_2_*_d_*); c) twist (*S*_4_); d) deckchair (*C*_2_*_h_*).

The ideal *D*_4_*_h_* symmetry, exhibiting the phenyl rings perpendicular to the planar central macrocycle (Figure 1a), does not correspond to the equilibrium state for the free FeTPP molecule. By rotating these peripheral rings, symmetry reductions down to *S*_4_, *D*_2_*_d_* or *C*_2_*_h_* could be obtained with energies lower than that of the *D*_4_*_h_* conformation. The central macrocycle is slightly deformed in the twisted conformation (*S*_4_) (Figure 1c) or more significantly deformed in the saddle (*D*_2_*_d_*) (Figure 1b) or deckchair (*C*_2_*_h_*) shape (Figure 1d). In fact, the *D*_4_*_h_* conformation corresponds to an average of these three conformations. The saddle conformation (*D*_2_*_d_*) has the lowest energy, while the twisted conformation (*S*_4_) is higher in energy by 0.04 eV. The *C*_2_*_h_* conformation is intermediate with an energy of 0.02 eV higher than *D*_2_*_d_*. Two magnetic states were found for each of these conformations in our calculations. The ground state of these three conformations is HS, the IS state being higher by 0.11 eV for *S*_4_, and by 0.04 eV for *D*_2_*_d_* and *C*_2_*_h_* (Table 1). Note that the IS state was found as ground state in some other calculated results [6–7]. The difficulty for obtaining a proper description of the fundamental state of Fe porphyrin is well known. This is a result of the competition between electron correlation, spin–orbital coupling and the on-site Coulomb repulsion [13].

**Table 1:** Relative energy, *E*_0_, and magnetic moment, μ, of *S*_4_, *D*_2_*_d_* and *C*_2_*_h_* conformations in the states *S* = 1 and *S* = 2. The reference energy is that of the HS *D*_2_*_d_* conformation.

	twist (*S*_4_)	saddle (*D*_2_*_d_*)	deckchair (*C*_2_*_h_*)

HS (*S* = 2), *E*_0_ (eV)	0.04	0	0.02
μ (μ_B_)	3.94	3.94	3.94
IS (*S* = 1), *E*_0_ (eV)	0.15	0.04	0.06
μ (*μ*_B_)	2.03	2.03	2.03

**Figure 2:**
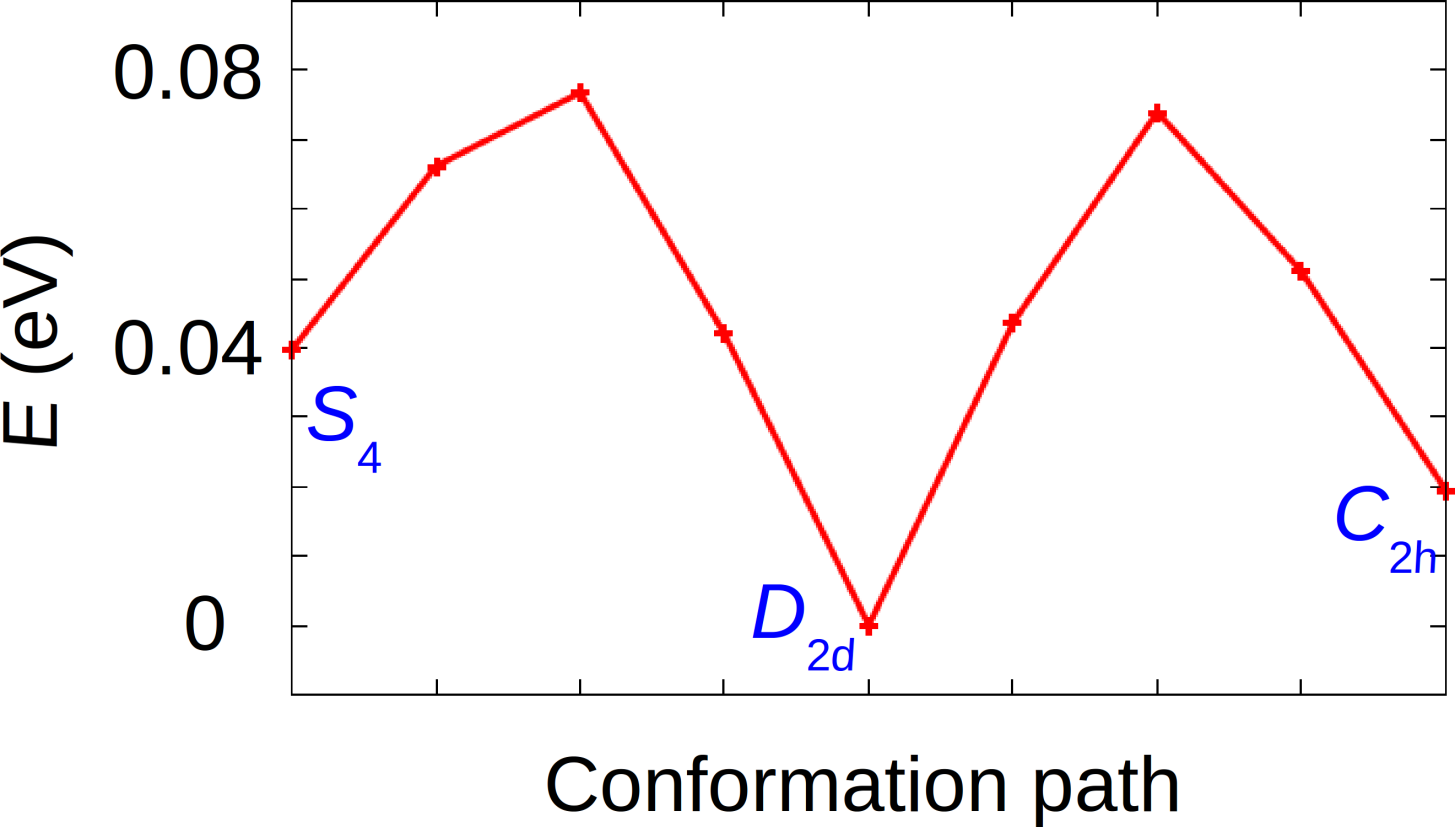
Activation energy to pass from the twist form (*S*_4_) to the saddle shape (*D*_2_*_d_*) and from the saddle shape to the deckchair conformation (*C*_2_*_h_*).

The activation barriers to switch between the three non-ideal conformations have been calculated (free molecules in vacuum) by using the nudged elastic band (NEB) method [14–15] (Figure 2). The barriers between the different conformations are 0.037 eV from *S*_4_ to *D*_2_*_d_*, 0.077 eV from *D*_2_*_d_* to *S*_4_, 0.074 eV from *D*_2_*_d_* to *C*_2_*_h_* and 0.055 eV for *C*_2_*_h_* to *D*_2_*_d_*.

### Intermolecular interactions

**Figure 3:**
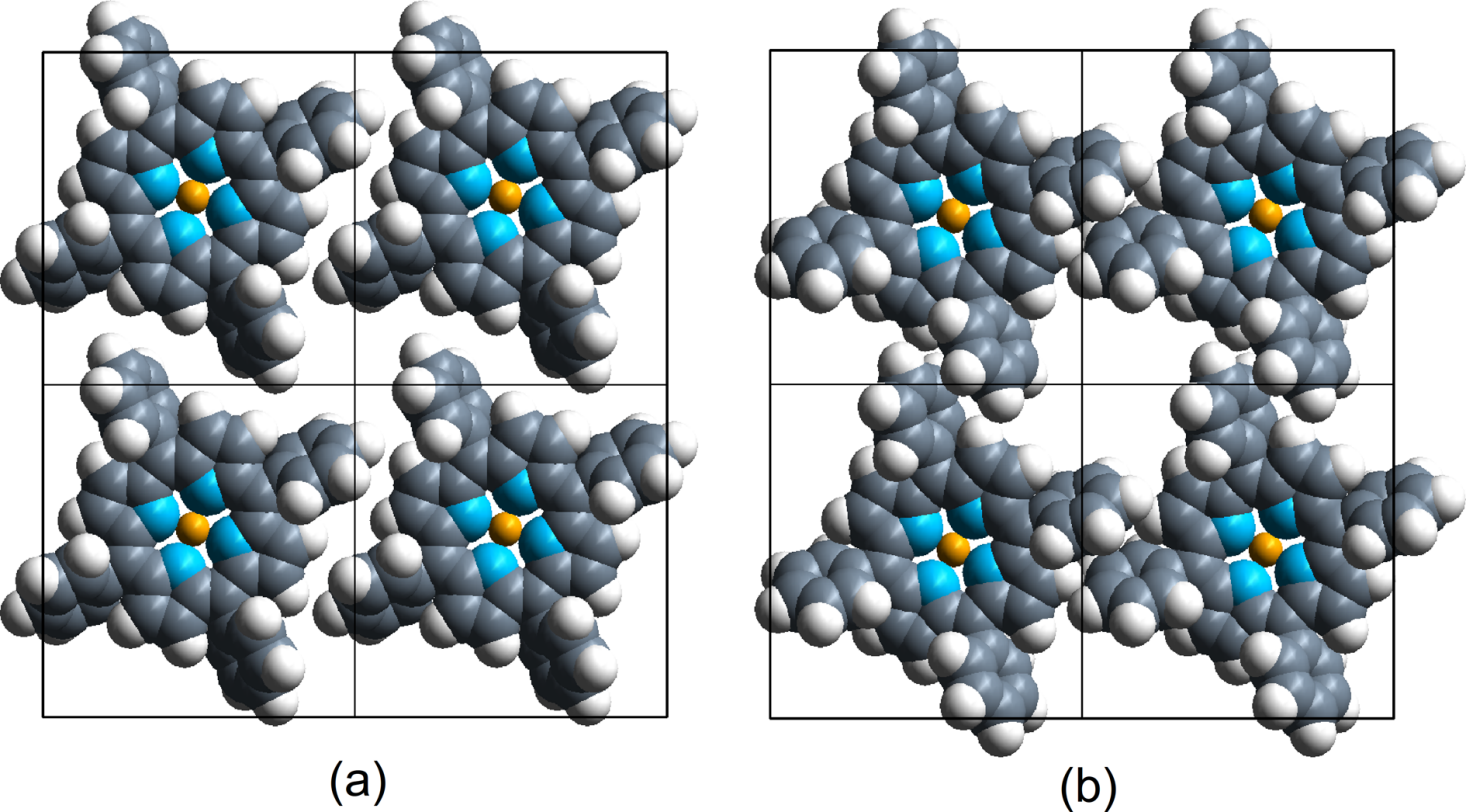
a) T-type (saddle conformation) and b) π–π-type (deckchair conformation) arrangements of FeTPP in 2D assemblies.

The *D*_2_*_d_* conformation is the most extensively studied for both the single molecule and molecules in self-assembled 2D islands [16–18]. In these assemblies, FeTPP forms a close-packed arrangement involving T-type interactions, i.e., the four phenyl rings of one molecule point their extremities perpendicularly towards the phenyl of the neighbouring FeTPP molecules (Figure 3a). In this configuration the central macrocycle has a saddle shape conformation (*D*_2_*_d_*) with the H atoms of two pyrrole groups (along the axis perpendicular to the figure) pointing upward and the H atoms of the other two pyrrole groups (along the horizontal axis in the plane of the figure) pointing downward (Figure 1b). In a less close-packed arrangement, the interaction between neighbouring FeTPP molecules is of π–π type. In the latter, all phenyl rings of a FeTPP molecule are parallel to the phenyl groups of neighbouring molecules (Figure 3b). The H atoms of two pyrrole rings (along the axis perpendicular to the figure) remain nearly in the same plane, while in one pyrrole group (the left one along the horizontal axis in the plane of the figure), the H atoms point upward and the H atoms of the pyrrole group at the opposite side (the right one along the horizontal axis in the plane of the figure) point downward (Figure 1d). In this second case, the macrocycle adopts a deckchair form (*C*_2_*_h_*). These conformations have been identified in sub-molecular resolution STM images on a Au(111) surface as shown in the work of N. Lin et al. [16] for the saddle conformation (twofold symmetry) and in the work of Gopakumar et al. [11] for the planar conformation (fourfold symmetry). The distance between the Fe centre of neighbouring molecules is about 14 Å in both cases and is consistent with a commensurate epitaxial mesh of (5 0; 3 6) on the Au(111) surface. T-type and π–π-type arrangements could also be distinguished by comparing the size of the void spaces between the molecules and their relative orientation as suggested in [18].

### Activation barrier between magnetic states

**Figure 4:**
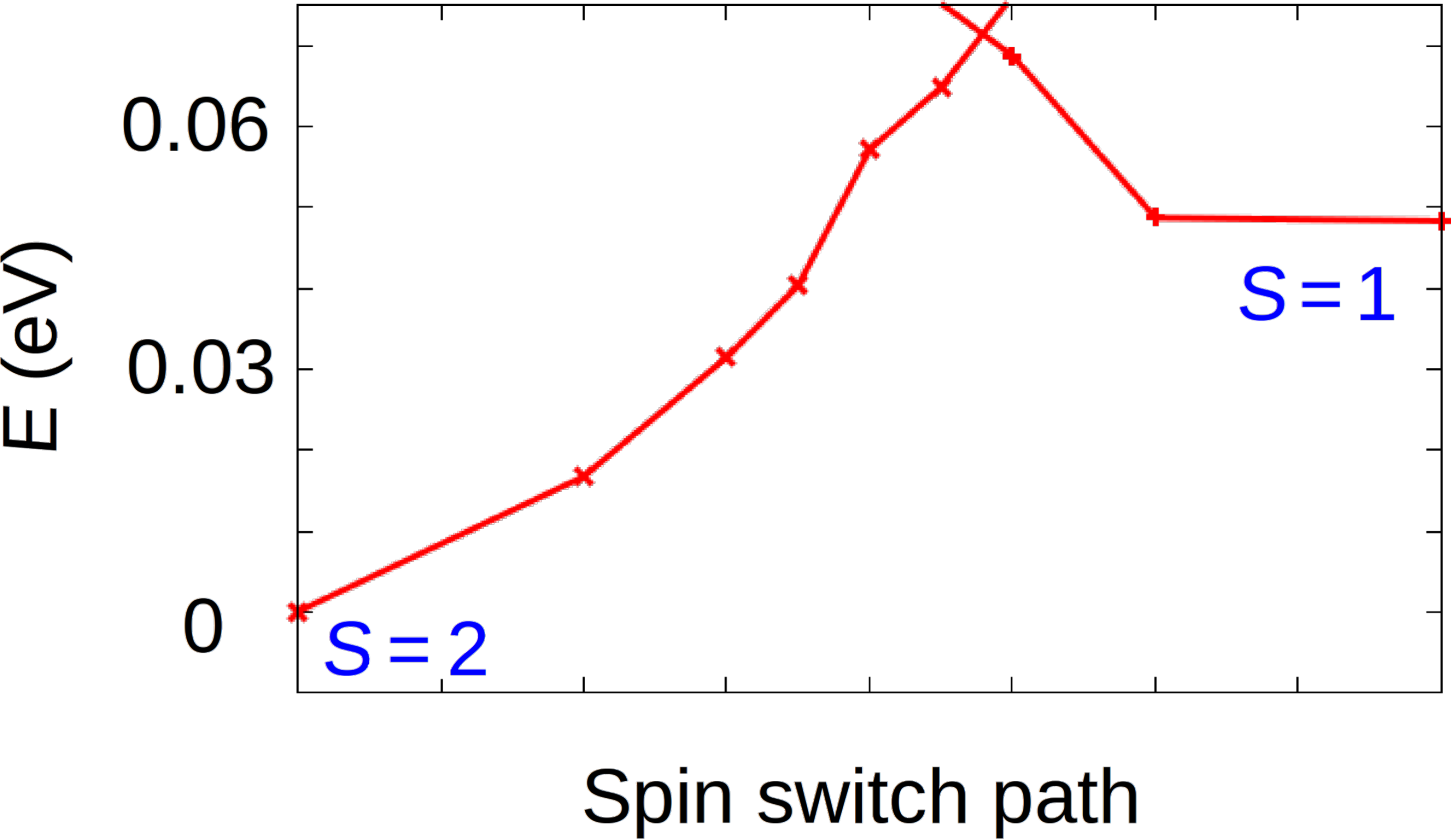
calculated activation barrier between HS (S = 2) and IS (S = 1) of a FeTPP molecule in *C*_2h_ conformation.

The saddle-shape conformation (*D*_2_*_d_*) has already been extensively reported in the literature [16–19]. Hence, we focus on the deckchair conformation (*C*_2_*_h_*) (Figure 3b). In order to evaluate the activation barrier of the free molecule between HS and IS states of this conformation, the energy of a series of intermediate images has been calculated (Figure 4). From these calculations, the activation energy from IS to HS (respectively, from HS to IS) was found to be 0.02 eV (respectively, 0.07 eV) with a Fe–N bond length of 2.059 Å in HS and 2.003 Å in IS. This is in accordance with the expected trend of a larger Fe–N distance in HS than in IS [2]. For the transition state, this length is about 2.026 Å. Therefore, for a free FeTPP molecule in *C*_2_*_h_* conformation, the identification of this transition state clearly confirmed the existence of two stable states, HS and IS.

### Adsorption configurations and spin states on Au(111)

**Figure 5:**
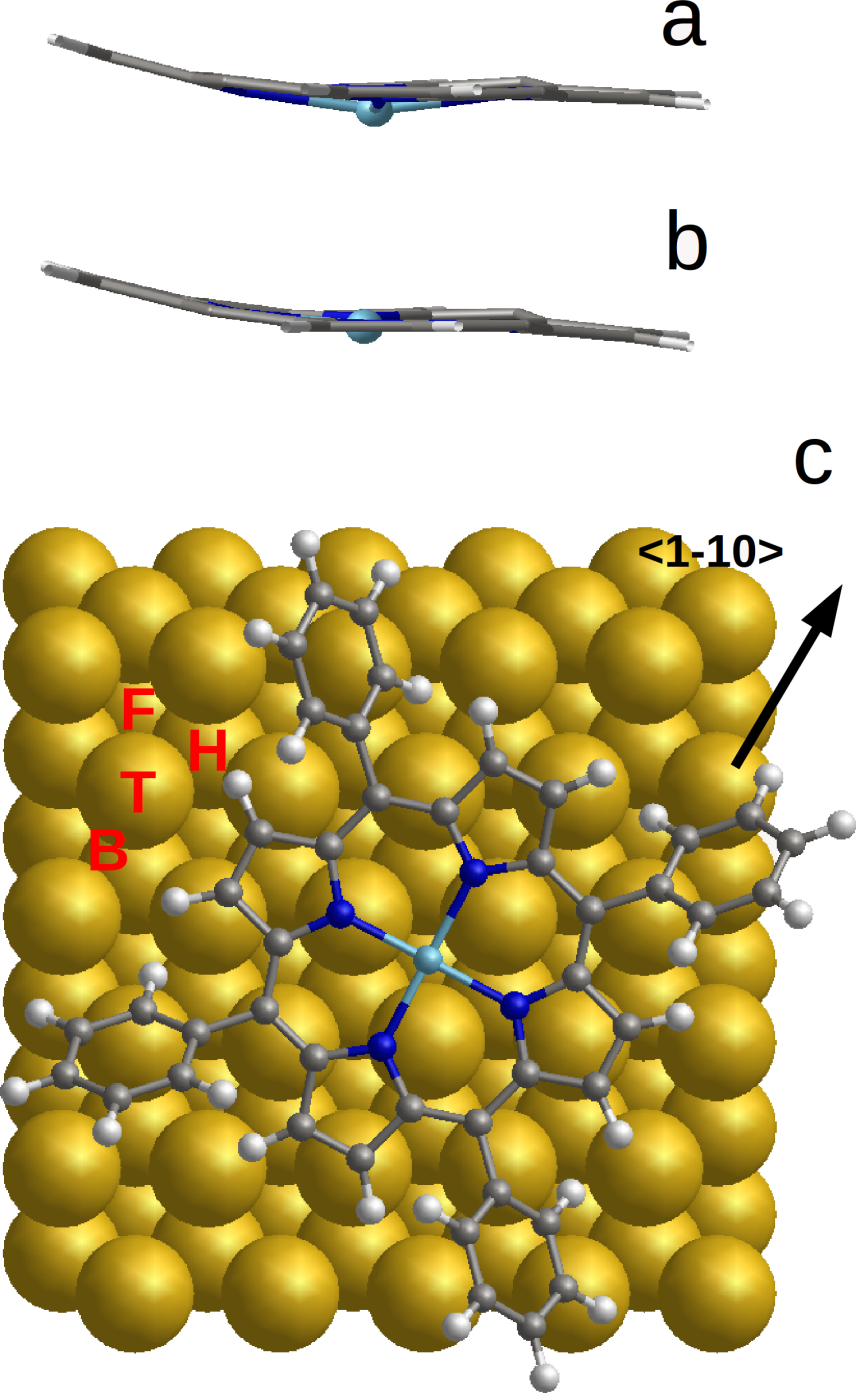
Conformation of the central porphyrin core in a) HS state; b) IS state (phenyl rings were omitted for clarity). In c) example of FeTPP adsorbed on fcc site of Au(111) on which the positions of the top (T), bridge (B), fcc (F) and hcp (H) sites are indicated. The optimized molecule is oriented along the <1−10> direction.

The deckchair form (*C*_2_*_h_*) molecule adsorbed on Au(111) also presents two magnetic states (HS, *S* = 2 and IS, *S* = 1) (Table 2). The most stable adsorption site is hollow-fcc in the HS state, which will be used as reference for the total energy comparison from now on. At the three other adsorption sites (hollow-hcp, top and bridge), the energy is higher by 0.04 eV. The magnetic moment of HS state is 4.18 ± 0.05 μ_B_ for these four sites. At hollow-fcc, the IS state energy is found to be higher by 0.07 eV than the HS state, while at the three other sites (hollow-hcp, top and bridge) the IS energies are 0.10 eV larger than the reference. The magnetic moment of the IS state on these sites is 2.24 ± 0.02 μ_B_. The molecule–surface distance is defined as the difference between the average *z*-values of C and N atoms in the macrocycle of FeTPP and the average *z*-values of the Au atoms of the top layer of the slab. This distance is 3.63 ± 0.06 Å independent of the magnetic state. However, the Fe-to-surface distance, *d*_Fe-surface_, is significantly different. In the HS state this distance is 3.17 ± 0.03 Å, and in the IS state this distance is 3.48 ± 0.04 Å. This difference indicates a stronger attractive interaction between the Fe atom and the surface in the HS state. The Fe–N bond length, *d*_Fe-N_, is slightly increased to 2.075 ± 0.002 Å in the HS state (respectively, slightly decreased to 2.002 ± 0.002 Å in the IS state) by comparison with the free molecule bond length. Furthermore, the side view of the central porphyrin macrocycle of the HS shows a deformation with the Fe atom pointing out of the plane formed by the four N atoms of the pyrrole rings (downwards to the surface) (Figure 5a). This deformation creates a square-based pyramid environment for the coordination sphere around the Fe atom, which also favours the HS state.

**Table 2:** Relative energy, *E*_0_, magnetic moment, μ, and Fe–surface distance, *d*_Fe-surface_, of FeTPP (in deckchair conformation) adsorbed on Au(111) surface in the HS and IS states. The reference is HS at the hollow-fcc site.

	top	bridge	hollow-fcc	hollow-hcp

HS (*S* = 2), *E*_0_ (eV)	0.04	0.04	0.00	0.04
μ (μ_B_)	4.23	4.13	4.13	4.13
*d*_Fe-surface_ (Å)	3.15	3.20	3.14	3.17
IS (*S* = 1), *E*_0_ (eV)	0.10	0.10	0.07	0.10
μ (μ_B_)	2.26	2.24	2.22	2.23
*d*_Fe-surface_ (Å)	3.44	3.52	3.43	3.46

**Figure 6:**
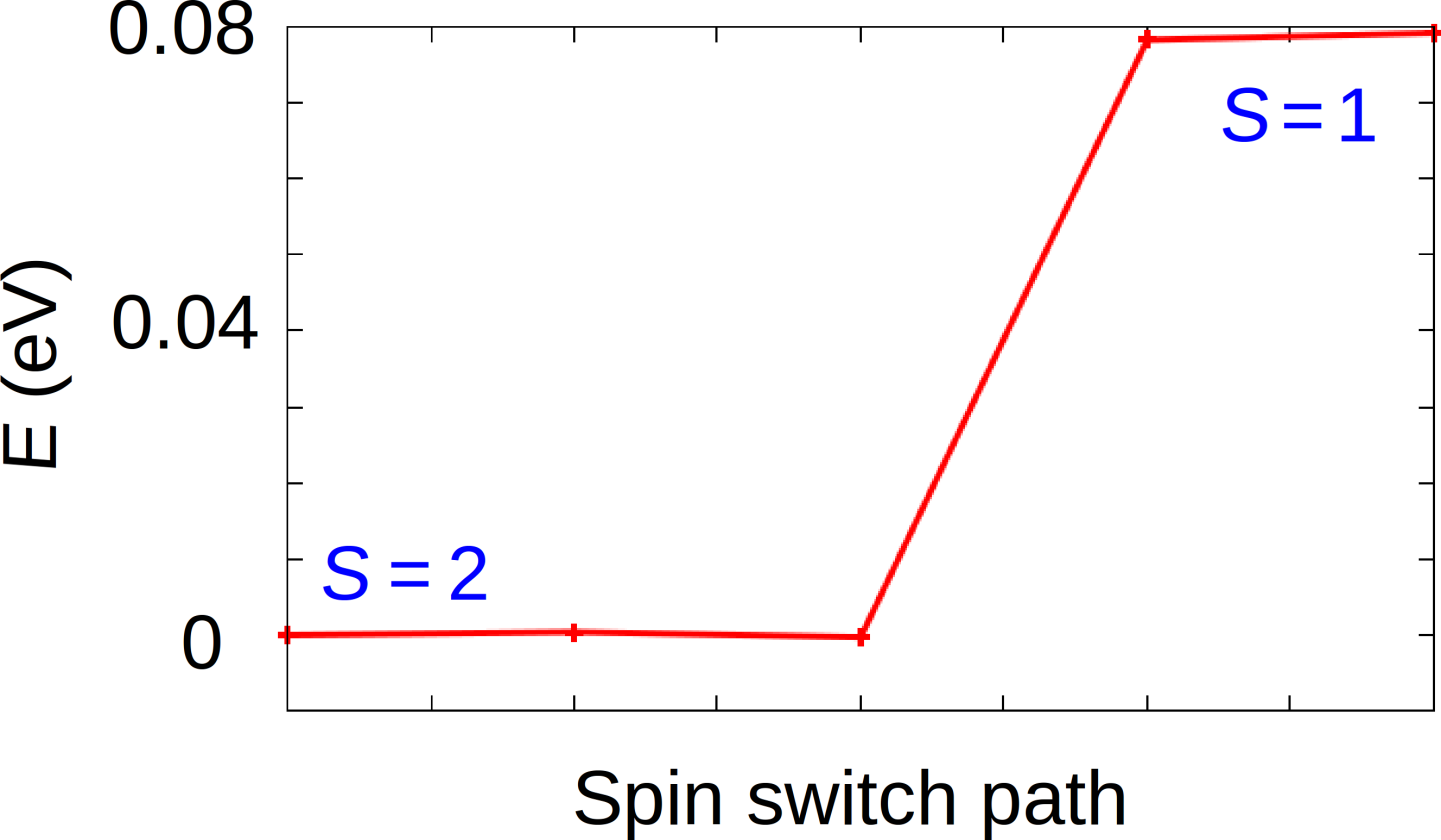
Calculated activation barrier between HS (*S* = 2) and IS (*S* = 1) of FeTPP (*C*_2_*_h_* conformation) adsorbed at the hollow-fcc site of Au(111).

The activation energy between the HS and IS states of FeTPP adsorbed on Au(111) has been evaluated. To do so, the NEB method was employed for FeTPP adsorbed on fcc site from HS (*S* = 2) to IS (*S* = 1) (Figure 6). While the activation energy from HS to IS (energy difference) is found to be 0.08 eV, no energy barrier was found to pass from IS to HS, indicating that the IS state is unstable when the molecule is adsorbed on Au(111). The fact that IS state was obtained during the geometrical optimization can be explained by a very flat potential energy surface (PES) around this configuration or by an activation barrier that is too small to be identified with the computational precision we used. Based on these results, the HS seems to be the only stable state for the adsorbed FeTPP in the deckchair conformation (*C*_2_*_h_*). In the following we check the strength of different interactions involved in the adsorption of this HS FeTPP on the Au(111) surface as well as the charge transfer, work function modification and the projected density of states (PDOS) variations.

In the reference configuration (HS, fcc), the adsorption energy is calculated to be −1.86 eV while the vdW contribution is found to be −1.70 eV. This small energy difference confirms the physisorption of FeTPP on Au(111). The molecule–surface distance of 3.63 ± 0.06 Å is consistent with the presence of the four peripheral phenyl rings acting as spacer that mitigate the coupling between the central macrocycle and the surface. X-ray standing wave measurements (XSW) on 3,4,9,10-perylene tetracarboxylic dianhydride (PTCDA) and on diindoperylene (DIP) on Au(111) report distances slightly lower (3.27 Å and 3.22 Å, respectively) [20–21]. The deformation energy of the adsorbed FeTPP is found to be +1.08 eV (0.03 eV for the gold surface), while the intramolecular vdW energy is −3.12 eV.

The charge transfer defined as the difference between the number of valence electron in the adsorbed molecule and in the free molecule in vacuum, Δ*q* = *q*_adsorbed molecule_ − *q*_free molecule_, was investigated through a Bader charge analysis [22]. The FeTPP molecule is positively charged by transferring 0.24*e* from the molecule to the Au(111) surface at the hollow-fcc site, but also at the bridge and hollow-hcp sites. At the top site, due to the direct coordination between the Fe and the Au atom, the transfer is slightly larger with 0.29*e*. The variation of partial charge on Fe (Δ*q*_Fe_) is a loss of about 0.04*e* (0.06*e* for HS on top site). The sign of this variation is the same as the molecule–surface charge transfer.

**Figure 7:**
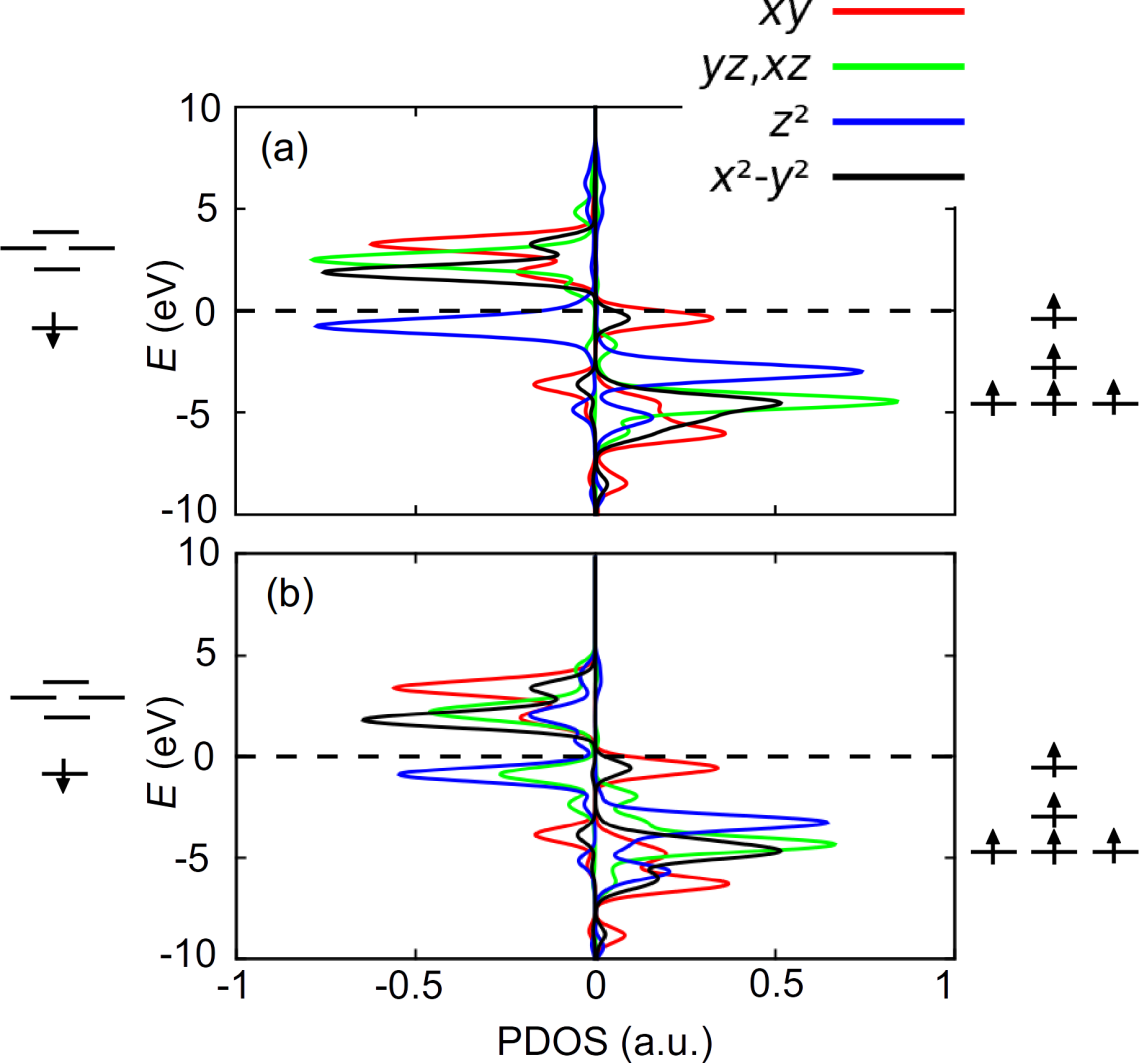
Spin-resolved PDOS on d-orbitals of the Fe atom of HS FeTPP (a) at the fcc site of Au(111) and (b) in the free molecule.

As shown in [16], the Fe centre contributes to electronic states around the Fermi level. We compare here the spin-resolved density of states projected (PDOS) onto the d-orbitals of Fe in the HS state before and after adsorption (with FeTPP adsorbed at the fcc site of Au(111) surface) (Figure 7). In these cases, only one orbital (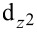) is doubly occupied (by both majority and minority spin). The four other orbitals (d*_xy_,* d*_xz_,* d*_yz_* and 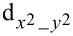) are occupied by majority spin, but unoccupied by minority spin. These four singly occupied orbitals confirm the spin multiplicity of *S* = 2 for the HS state. As displayed in Figure 7, the charge transfer from FeTPP to the Au(111) surface does not disturb the distribution of d-orbitals around the Fermi level.

The work function Φ = *E*_pot_ − *E*_Fermi_ on uncovered Au(111) was calculated with *E*_pot_ being the local potential at the middle of vacuum of the simulation cell. The evaluated value of 5.14 eV is in good agreement with the experimental one (5.35 eV) [23]. After the adsorption, the work function was reduced to 4.19 eV. This value is consistent with that measured on copper phthalocyanine (CuPc) adsorbed on Au(111) [24].

### Adsorption on Ag(111) and Cu(111) surfaces

**Figure 8:**
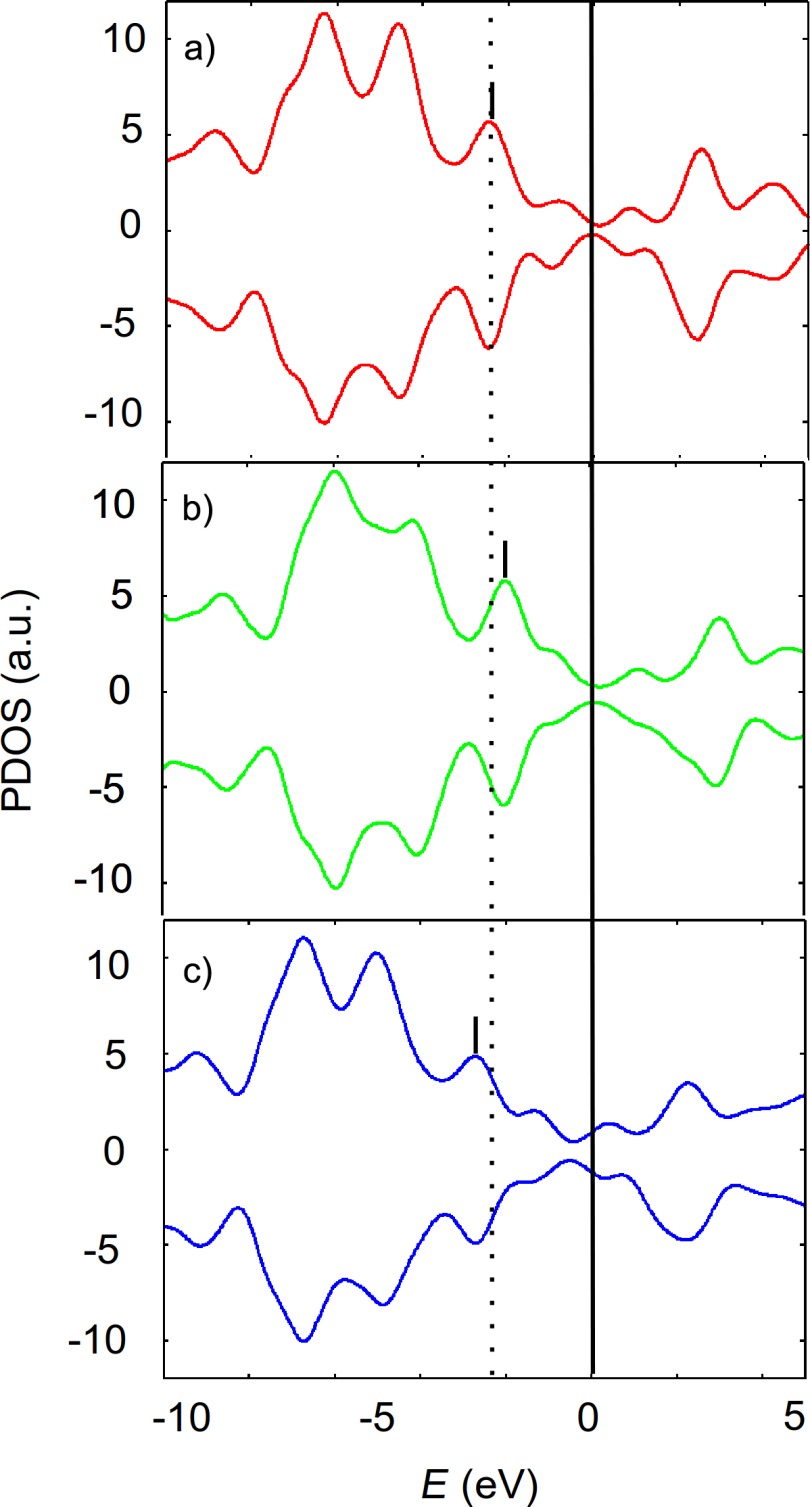
Spin-resolved PDOS of FeTPP. a) free molecule (red), adsorbed on fcc site of b) Au(111) (green), c) on Ag(111) (blue). The shift of PDOS on Cu(111) is similar to that on Ag(111).

It is well known for large organic molecules such as PTCDA or DIP that their interaction with coinage-metal surfaces are different and the binding strength increases in the form of Au < Ag < Cu while the molecule–surface distance decreases [20–21]. In order to verify this trend for FeTPP, calculations were performed on fcc, hcp, top and bridge sites of Ag(111) and Cu(111) surfaces (Figure 8).

The adsorption energy for HS FeTPP on Ag(111) is −4.99 ± 0.01 eV with a molecule–surface distance of *d*_FeTPP-Ag(111)_ = 3.06 ± 0.01 Å and an Fe–surface distance of *d*_Fe-Ag(111)_ = 2.82 ± 0.01 Å. On Cu(111), the adsorption energy of HS FeTPP is −4.85 ± 0.02 eV with a molecule–surface distance of *d*_FeTPP-Cu(111)_ = 2.82 ± 0.05 Å and an Fe–surface distance of *d*_Fe-Cu(111)_ = 2.48 ± 0.04 Å. The adsorption energies on these two surfaces are significantly larger than those on Au(111). The main reason is the molecule–surface vdW contribution, which is overestimated [25] on Ag(111) and Cu(111) (−6.32 eV and −6.42 eV, respectively when adsorbed on fcc site). As mentioned in the computational details, the C_6_ parameter for Au was optimized on a model system in such a manner that it represents about one third of the standard value. For calculations on Ag(111) and Cu(111), we did not optimize this parameter. As the weak molecule–surface interaction on Au(111) is sufficient to stabilize the HS state of the adsorbed FeTPP molecule, we did not expect a contrary result on Ag(111) and Cu(111) surfaces.

The Bader charge analyses result in a negatively charged FeTPP molecule on Ag(111), with 0.60 ± 0.02 electron being transferred from the surface to the molecule in the HS state. The Fe centre has only gained (0.02 ± 0.01)*e*. This charge variation does not modify the magnetic state of the adsorbed molecule. The same trend was observed on Cu(111), with an average of (0.66 ± 0.08)*e* being transferred from the surface to the molecule in HS state, and 0.04 electron gain on the Fe centre.

Upon FeTPP adsorption, the work function of the Ag(111) surface (respectively, Cu(111) surface) is found to be reduced from 4.41 eV (4.77 eV) for the bare substrate to 3.71 eV (4.36 eV) for the covered surface. The same trends were reported for the adsorption of benzene on Ag(111) [26] and on Cu(111) [23]. From this study, we can conclude that all three tested (111) surfaces exhibit the same trend of a lowered work function upon molecular adsorption. Nevertheless, we have found that the charge transfer on Au(111) occurred in opposite direction than that on Cu(111) and Ag(111). This result is perfectly reflected in the comparison of the PDOS of the free molecule and of the adsorbed molecule. By adsorbing the FeTPP molecule on Au(111), the occupied electronic states of the molecule are shifted towards the Fermi level, thus facilitating the charge transfer from the molecule to the substrate. By adsorbing on Ag(111) and Cu(111), the unoccupied molecular states are shifted towards the metal Fermi level, thus facilitating the charge transfer from the substrate to the molecule. These shifts explain the direction of the charge transfer.

In summary, by performing spin-polarized DFT and NEB calculations, we have identified two stable magnetic states of the free FeTPP molecule in its deckchair conformation (*C*_2_*_h_*). The two states (HS and IS) were separated by an activation barrier of 0.07 eV to pass from *S* = 2 to *S* = 1 and an activation barrier of 0.02 eV to pass from *S* = 1 to *S* = 2. However, when this molecule is adsorbed on Au(111) in a monolayer, the IS state is no longer stable as the activation barrier from *S* = 1 to *S* = 2 disappears. The most stable magnetic state on Au(111), HS, has an adsorption energy of −1.83 ± 0.02 eV with a contribution of vdW interactions of −1.70 eV, and the central porphyrin macrocyle is at a distance of 3.63 ± 0.06 Å above the surface. These physisorption characteristics were confirmed by a small charge transfer (0.24*e*) from the molecule to the surface. By changing from Au(111) to Ag(111) and to Cu(111) surfaces, the adsorption energy increases and the molecule–surface distance decreases as expected. However, these quantities cannot be compared quantitatively since we have used an optimized C_6_ parameter for Au and standard DFT-D2 parameter for Ag and Cu. Qualitatively, the amount of charge transfer was slightly increased to 0.60*e* from Ag(111) to FeTPP and to 0.64*e* from Cu(111) to the molecule. In spite of these charge transfers, the partial charge on the Fe centre remains almost unchanged, i.e., 0.1*e* on Au(111), 0.02*e* on Ag(111) and 0.04*e* on Cu(111). Due to molecular adsorption, the work function of Au(111) was reduced by 0.95 eV, this reduction being 0.71 eV on Ag(111) and 0.41 on Cu(111).

## Conclusion

In this study, we have focused our investigation on the deckchair conformation (*C*_2_*_h_*) of the FeTPP molecule, which favours π–π-type interactions in self-assembled monolayers on Au(111), Ag(111) and Cu(111) surfaces. In spite of the presence of two stable magnetic states in the free molecule, only the high-spin (*S* = 2) state is stable when adsorbed on metal. These results show that the physisorption of FeTPP on coinage metal surfaces is strong enough to modify the ligand-field environment of Fe. This result reveals that an external permanent element such as a STM tip or an additional molecule is needed to use FeTPP or similar molecules as model system for molecular spin switches.

## Method and Computational Details

Spin-polarized DFT calculations were performed using a slightly modified version of the Vienna ab initio simulation package VASP [27–30]. Projector-augmented wave (PAW) pseudo-potential [31–32], as well as the exchange–correlation functional proposed by Perdew–Burke–Ernzerhof (PBE) in the framework of the generalized gradient approximation [33–34] were employed. The van der Waals dispersive interaction correction according to Grimme's DFT-D2 method [35] was considered for inter- and intra-molecular interactions as well as molecule–surface interactions. The C_6_ parameter was optimized in a model system on Au(111) in such a manner that its value is about three times smaller than the standard C_6_ [36]. This smaller value is due to the screening effect similar to that demonstrated by Tkatchenko et al. [37]. In addition, the DFT-D2 correction was not applied in the metal slabs as no significant improvement has been demonstrated [37–38]. The kinetic cut-off energy for the plane-waves basis was set to 410 eV for Au, 460 eV for Ag and 500 eV for Cu. For the FeTPP/Au(111) system, a simulation supercell of (14.76 Å × 15.33 Å × 27.51 Å) containing 120 Au atoms (30 atoms × 4 layers) and 77 atoms for the Fe-TPP molecule (C_44_H_28_N_4_Fe) was used. This dimension is (14.73 Å × 15.30 Å × 27.45 Å) for Ag(111) and (17.81 Å × 15.42 Å × 25.18 Å) for Cu(111) for which each atomic layer contains 48 atoms (4 × 48 = 192 Cu atoms in the slab). The Brillouin zone sampling in reciprocal space was restricted to the Γ point. The validity of this restriction was tested on the reference configuration (FeTPP at the hollow-fcc site of Au(111)) with a (5 × 5 × 1) Monkhorst–Pack *k*-point mesh. This comparison gives a difference lower than 1% for the total energy and the bond length. The difference in density of states and local electrostatic potential (including dipole interaction correction) are not noticeable. DFT + U method as proposed by Dudarev [39] was used to take into account the on-site d-electron correlation of the central Fe atom. For this purpose, the Coulomb repulsion parameter *U* was set to 4 eV and the exchange parameter *J* was set to 1 eV (as in [11]). The convergence condition of the self-consistent electronic loops was set to 10^−6^ eV, while the atomic positions were relaxed until the forces reached a value lower than 0.01 eV/Å. The atoms of the two bottom layers of the metal slab were kept fixed at their bulk positions and all other atoms were allowed to relax without any constraint. The adsorption energy was determined as *E*_ads_ = *E*_tot_ − *E*_slab_ − *E_mol_* where *E*_tot_ is the total energy of the system containing a FeTPP molecule on a Au/Ag/Cu (111) slab, *E*_mol_ is the energy of the FeTPP molecule in vacuum and *E*_slab_ is the total energy of the 4-layer metal slab.
